# Rational Design of a Peptidomimetic Inhibitor of Gelsolin Amyloid Aggregation

**DOI:** 10.3390/ijms232213973

**Published:** 2022-11-12

**Authors:** Michela Bollati, Kaliroi Peqini, Luigi Barone, Carmina Natale, Marten Beeg, Marco Gobbi, Luisa Diomede, Michelangelo Trucchi, Matteo de Rosa, Sara Pellegrino

**Affiliations:** 1 Institute of Biophysics, National Research Council (IBF-CNR), c/o Department of Biosciences, University of Milano, Via Celoria 26, 20133 Milano, Italy; 2 Department of Pharmaceutical Science, “A. Marchesini” General and Organic Chemistry Section, University of Milano, Via Venezian 21, 20133 Milano, Italy; 3 Department of Molecular Biochemistry and Pharmacology, Istituto di Ricerche Farmacologiche Mario Negri IRCCS, Via Mario Negri 2, 20156 Milano, Italy

**Keywords:** amyloidosis, aggregation, peptidomimetics, gelsolin, *C. elegans*

## Abstract

Gelsolin amyloidosis (AGel) is characterized by multiple systemic and ophthalmic features resulting from pathological tissue deposition of the gelsolin (GSN) protein. To date, no cure is available for the treatment of any form of AGel. More than ten single-point substitutions in the *GSN* gene are responsible for the occurrence of the disease and, among them, D187N/Y is the most widespread variant. These substitutions undergo an aberrant proteolytic cascade, producing aggregation-prone peptides of 5 and 8 kDa, containing the Gelsolin Amyloidogenic Core, spanning residues 182–192 (GAC_182–192_). Following a structure-based approach, we designed and synthesized three novel sequence-specific peptidomimetics (LB-5, LB-6, and LB-7) built on a piperidine-pyrrolidine unnatural amino acid. LB-5 and LB-6, but not LB-7, efficiently inhibit the aggregation of the GAC_182–192_ amyloidogenic peptides at sub-stoichiometric concentrations. These peptidomimetics resulted also effective in vivo, in a *C. elegans*-based assay, in counteracting the proteotoxicity of aggregated GAC_182–192_. These data pave the way to a novel pharmacological strategy against AGel and also validate a toolbox exploitable in other amyloidogenic diseases.

## 1. Introduction

Familial amyloidosis Finnish type (FAF), also known as AGel amyloidosis (AGel) is a rare autosomal dominant disease characterized by specific systemic and ophthalmic features. AGel most notably results in sight-affecting corneal dystrophy, chronic corneal ulceration [1]; a high incidence of cutis laxa, dermatochalasis, and facial nerve palsies; cardiac conduction abnormalities and renal complications [2], and rare reports of nephrotic syndrome [3]. The reported symptoms stem from single-point mutations in the gelsolin (GSN) protein, which lead to the deposition of GSN amyloid fibrils in multiple organs and tissues.

GSN is a calcium-dependent protein, which has a role in multiple biological processes, being responsible for the assembly and disassembly of actin filaments [4]. The protein is organized into six homologous domains (named G1–G6) that share the same GSN-like fold [5]. Several different amyloidogenic variants of GSN have been identified over the years [6,7,8,9,10,11,12,13]. Most of them cluster within two specific regions of the protein: (i) the calcium-binding site of the second domain (G2) and (ii) the interface between domains four and five (G4:G5). Substitutions in these two different hot spots likely trigger two different aggregation mechanisms.

The most common forms of AGel are caused by the substitutions in the G2 domain, including D187N/Y, N184K, and G167R mutations (numbering according to the mature plasma form of the protein). G2-linked substitutions lead to a local destabilization and increased flexibility of the domain, thus triggering the exposure of an otherwise buried sequence, which is aberrantly cleaved by furin in the Golgi [14,15,16]. The larger product of furin activity, the C68 fragment, undergoes another proteolytic event that eventually leads to the production of two aggregation-prone peptides of 5 and 8 kDa [17]. These peptides readily aggregate because they contain an amyloidogenic core sequence, called Gelsolin Amyloidogenic Core (GAC), identified in systematic studies as spanning residues 182 to 192 [18] or 187 to 193 [19] of GSN. Other larger analogs harbouring GAC, such as a 15-mer [20] and the isolated G2 domain [21], were shown to recapitulate pathological GSN aggregation in vitro.

We have recently demonstrated that the G4:G5-linked variants, namely A551P, E553K, and M517R, are not susceptible to furin proteolysis and are endowed with amyloid aggregation propensity in their full-length form [22]. Other yet-to-be-identified amyloidogenic stretches of the protein may be responsible for this proteolysis-independent aggregation pathway.

To date, no specific pharmacological therapy for any form of the disease is available, although a few strategies have been explored, such as those based on therapeutic nanobodies [23,24]. Treatment of this amyloidosis is solely based on the amelioration of symptoms through surgery, transplantation, and other invasive medical procedures. The inhibition of the aggregation of GSN in the form of aggregation-prone fragments or the full length, to prevent the deposition of large aggregates and the circulation of highly toxic soluble oligomers can thus be targeted to slow down or even block pathological aggregation of GSN. A few small molecules such as non-selective polyphenols, phospholipids, and other repurposed drugs [19,20,25] were shown to modulate GSN aggregation in vitro and counter the associated toxicity.

We here propose a new strategy, based on the use of novel selective peptidomimetics able to interfere with the formation of amyloidogenic GSN aggregates. Peptidomimetics are indeed a profitable class of compounds designed to mimic bioactive peptides and/or protein domains and are often characterized by improved biological and pharmacokinetic properties [26,27,28,29]. The binding of the here-developed peptidomimetics to the GAC sequence, by mimicking the interactions with the flanking sequences, can avoid the aberrant protein-protein interactions underlying the aggregation process and preserve the monomeric state.

## 2. Results

### 2.1. Sequence- and Structure-Based Design of the Peptidomimetics and Their Synthesis

**Figure 1:**
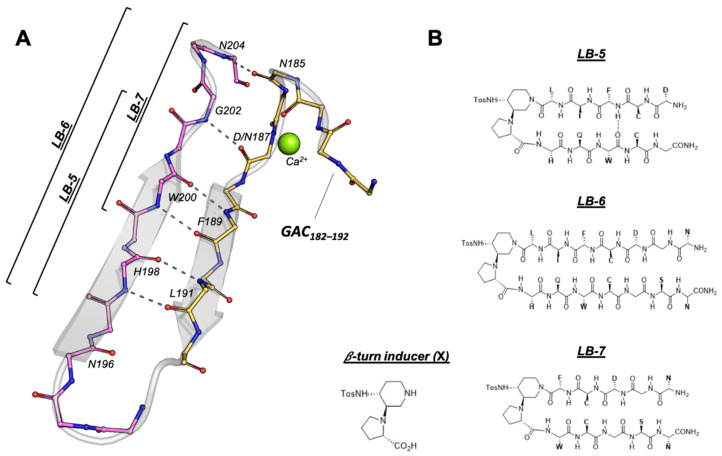
Design and structure of the LB peptidomimetics. (**A**) Backbone conformation and localization of the GAC_182–192_ in the context of the native 3D structure of GSN G2 (pdb id 6QW3 [32]). The flanking sequence spanning residues 194–204 used as a template to design the peptidomimetics is also shown. (**B**) Chemical structure of the peptidomimetics LB-5, LB-6, and LB-7 and of the β-turn inducer.

To design novel selective peptidomimetics able to interfere with the formation of amyloidogenic aggregates in tissues of AGel patients, we exploited the recently developed approach that successfully blocked fibrils formation in Aβ aggregation and hIAPP [30,31]. The peptidomimetics were designed starting from the localization and the interactions of the amyloidogenic sequence, residues 182-SFNNGDCFILD-192 (GAC_182–192_) with flanking sequences (FS) in the native structure of the protein (Figure 1A). Among the others, the 194-GNNIHQWCGSN-204 (FS_194–204_) sequence showed an extended interaction area, providing a broader chemical space. Both GAC_182–192_ and FS_194–204_ adopt a β-strand conformation and interact with each other in an antiparallel fashion. The region comprising the L191-H198 and F189-W200 pairs shows a near-ideal beta structure where the two strands are tethered by 4 H-bonds (Figure 1A). The second half of the hairpin is loosely bound due to the distortion of the strands, although a disulphide bond between residues 188 and 200 may contribute to its stabilization. Three peptidomimetics, called LB-5, LB-6, and LB-7, were designed using sequences of variable lengths of GAC_182–192_ and FS_194–204_ and linking them with the synthetic piperidine-pyrrolidine scaffold **X** (Figure 1B). In particular, LB-6 covered the longest sequences of GAC_182–192_ (H_2_N-NGDCFIL**X**HQWCGSN-CONH_2_), LB-5 covered the C-terminus (H_2_N-DCFIL**X**HQWCG-CONH_2_) whereas LB-7 the central portion of GAC_182–192_ (H_2_N-NGDCF**X**WCGSN-CONH_2_). Harboring a portion of the amyloidogenic core, these molecules could have the potential to interact with the monomeric and/or oligomeric forms of GAC_182–192_ preventing further growth of the amyloid filament.

The GAC_182–192_ amyloidogenic core and the LBs peptidomimetics were synthesized by microwave-assisted solid phase peptide synthesis. This technique allows for obtaining, in a short amount of time, peptides with a high degree of purity and good yield [33]. The compounds were assembled using the Rink-Amide resin (0.69 mmol/g loading) as the solid support, and the DIC/oxima coupling system. The coupling of the scaffold was performed manually on the resin using HOBt/HBTU/DIEA (1.5/1.5/3 eq) as coupling reagents.

### 2.2. Peptidomimetics Inhibit GAC_182–192_ Aggregation

To evaluate the ability of LBs to affect the propensity of GAC_182–192_ to form amyloid fibrils, we carried out in vitro fluorescence Thioflavin T (ThT) assays. ThT is a commonly used probe specific for the visualization and quantification of amyloid-like structures. Upon binding to fibrils, ThT gives a strong fluorescence signal. Several long-known and emerging factors can promote and modulate aggregation [34,35], to identify optimal conditions for pH and GAC_182–192_ concentration preliminary tests were performed (Figure S1). As expected based on literature data on analogous peptides [17,18,20,36], GAC_182–192_ aggregation kinetics showed a concentration and pH (lower the faster) dependence, whereby the presence of a reducing agent had a minor impact. Based on these results, all the tests were then carried out for 96 h at 37 °C, under stirring conditions and pH 4.5, using a concentration of GAC_182–192_ of 100 µM.

**Figure 2:**
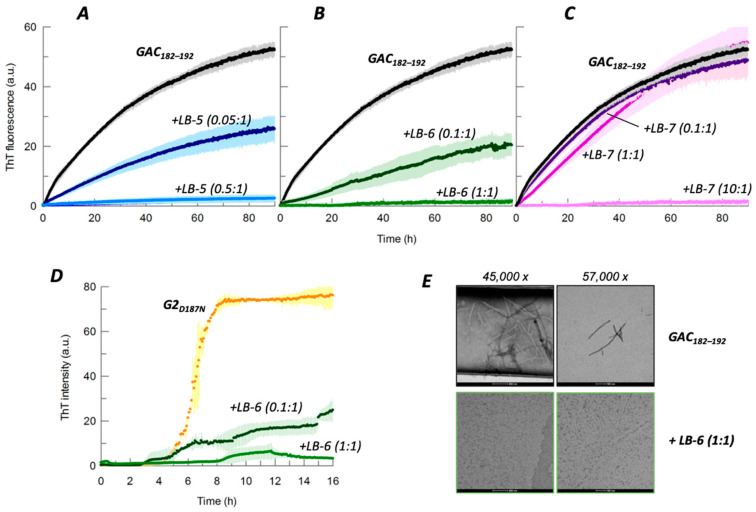
Inhibitory activity of peptidomimetics. (**A**–**C**) Representative curves of ThT fluorescence assays showing GAC_182–192_ (100 µM) aggregation over time in the absence (black curve) and presence of increasing concentrations of LB-5 (5–50 µM, blue trace), LB-6 (10–100 µM, green), or LB-7 (10–1000 µM, purple). Data are the mean ± SD (bars of a lighter shade of data colour) of three replicates. (**D**) Under the same conditions, the ability of LB-6 (7.5–75 µM) to inhibit aggregation of 75 µM isolated G2 carrying the D187N mutation (G2_D187N_) was evaluated. (**E**) Representative transmission electron microscopy images of 100 µM GAC_182–192_ incubated for 96 h in the absence or presence of 100 µM LB-6 (1:1).

The ThT fluorescence of GAC_182–192_ incubated alone increased rapidly along the 96 h experiment, demonstrating the propensity of this peptide to form amyloid fibrils (Figure 2A–C). Whereby, the ThT signal of single LBs alone did not increase during the time of the experiments (Figure S2), indicating that the peptidomimetics did not form amyloid-like structures. As shown in Figure 2, the co-incubation of 5 µM LB-5 (0.05:1 LB:GAC molar ratio) or 10 µM LB-6 (0.1:1) decreased by roughly 60% the end-point ThT fluorescence signal. The aggregation process was completely inhibited at 50 µM of LB-5 (0.5:1) and 100 µM LB-6 (1:1), respectively. On the contrary, LB-7 did not affect the end-point ThT fluorescence of GAC_182–192_ at the two lowest concentrations tested. Complete inhibition of aggregation was reached only at 1000 µM (10:1) LB-7.

To investigate whether the peptidomimetics can affect the morphology of the aggregated GAC_182–192_, transmission electron microscopy (TEM) analysis was done on GAC_182–192_ after a 96 h incubation in the absence or presence of LB-6. As shown in Figure 2E, after 96 h, the time corresponding to the plateau of ThT fluorescence curves, GAC_182–192_ existed as mature straight, and several micrometer-long, fibrils. In the presence of an equimolar concentration of LB-6, no fibrillar entities were observed.

LB-6 anti-aggregation activity was also evaluated under the same experimental conditions using the isolated G2 domain of GSN carrying the D187N mutation (G2_D187N_) instead of GAC_182–192_ (Figure 2D). At a 0.1:1 LB:G2 molar ratio, i.e., 7.5 µM, LB-6 reduced the end-point ThT fluorescence of G2_D187N_ by over 70% and, at 75 µM the ThT signal was completely inhibited.

### 2.3. LB-5 and LB-6 Counteract the Toxicity of Aggregated GAC_182–192_ In Vivo

The ability of the LB peptidomimetics to reduce the toxicity of GAC_182–192_ aggregates in vivo was investigated by employing the invertebrate *C. elegans*, a well-validated animal model able to recognize the toxic forms of amyloidogenic proteins, including different G2 and full-length GSN variants [22,23]. Thanks to their sensitivity to sublethal doses of chemical stressors, worms react to amyloid oligomers and/or aggregates by reducing the contraction and relaxation of the pharyngeal muscle, defined as “pumping rate”, providing a direct relationship between the protein structure and its toxicity [37,38,39,40]. The use of *C. elegans* as a biosensor is particularly useful for pre-clinical studies aimed at screening, rapidly and inexpensively, the activity of new drugs or small molecules.

**Figure 3:**
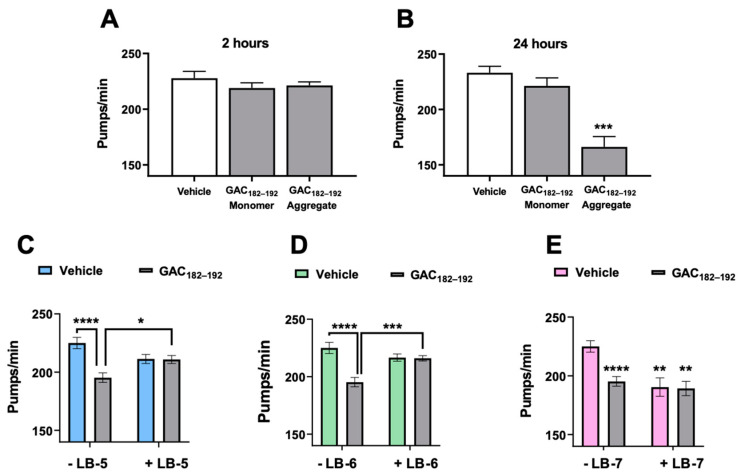
Effect of peptidomimetics on the toxicity caused by GAC_182–192_ in *C. elegans*. (**A**,**B**) GAC_182–192_ was dissolved at 100 µM in 200 mM sodium citrate buffer, pH 4.5, containing 100 mM NaCl. An aliquot was withdrawn immediately after the dissolution (Monomer) and 48 h after the incubation at 37 °C (Aggregate). Samples were then diluted in 10 mM phosphate buffered saline, pH 7.4, to the final concentration of 20 µM and administered to *C. elegans* (50 µL/50 worms). Control worms were treated in the same experimental condition with 10 mM phosphate buffered saline, pH 7.4 (50 worms/50 µL) (Vehicle). The pharyngeal activity was determined 2 and 24 h after treatment by determining the number of pharyngeal bulb contractions (pumps/min). Data are means ± SE (N = 10). *** *p* < 0.001 vs. Vehicle, one-way ANOVA, and Bonferroni’s post hoc analysis. (**C**–**E**) GAC_182–192_ (100 µM) in 200 mM sodium citrate buffer, pH 4.5, containing 100 mM NaCl, was incubated for 48 h at 37 °C in the absence or presence of 10 µM LB-5, LB-6 or LB-7. At the end of incubation, samples were diluted five times and 20 µM GAC_182–192_ + 2 µM peptidomimetic was administered to worms (50 worms/50 µL). Control worms were treated with 20 µM GAC_182–192_, 2 µM LB-5, LB-6, or LB-7 alone, or 200 mM sodium citrate buffer, pH 4.5 (50 worms/50 µL). The pharyngeal activity was determined 24 h after treatment. Data are means ± SE (N = 10). * *p* < 0.05, ** *p* < 0.005, *** *p* < 0.001, and **** *p* < 0.000.1, one-way ANOVA and Bonferroni’s post hoc analysis. Interaction GAC_182–192_/LB-5: *p* = 0.001, GAC_182–192_/LB-6: *p* = 0.0003, two-way ANOVA, and Bonferroni’s post hoc analysis.

First, we investigated whether GAC_182–192_, in the monomeric or aggregated state, can be recognized as toxic by *C. elegans*. To this end, the peptide was administered to worms before and 48 h after the incubation at 37 °C, under the same experimental conditions applied for ThT experiments. At this time-point, a significant increase of the ThT fluorescent signal was observed indicating the presence in solution of aggregated amyloidogenic forms of GAC_182–192_ (Figure 2). The pumping rate of nematodes was measured 2 and 24 h after the administration to evaluate the transient and permanent toxic effects, respectively. Interestingly, the pharyngeal activity of worms resulted significantly reduced only 24 h after the administration of the aggregated GAC_182–192_ indicating its ability to induce a permanent dysfunction. The peptide, in its monomeric form, did not exert any proteotoxic activity (Figure 3A,B).

We then evaluated whether the peptidomimetics, inhibiting the aggregation of the peptide, can also protect worms from pharyngeal dysfunction. LBs alone, at 2 or 20 µM, were administered to worms to determine their potential toxicity. No significant reduction of the pharyngeal dysfunction was detected in worms 24 h after the treatment with LB-5 or LB-6 at both concentrations whereas LB-7, already at 2 µM, resulted in toxicity (Figure S3). GAC_182–192_ at 100 µM was incubated for 48 h in the presence or absence of 10 µM LBs and, after dilution, were administered to worms (20 µM GAC_182–192_ and 2 µM peptidomimetics). Despite the toxic effect of LB-7, we decided to test its effect in vivo, too. As shown in Figure 3, the co-incubation of GAC_182–192_ with LB-5 and LB-6 completely counteracted the pharyngeal dysfunction caused in worms by the aggregated form of the peptide whereas LB-7, did not exert any protective activity.

## 3. Discussion

Gelsolin amyloidosis (AGel) is a rare disease characterized by the deposition of protein aggregates in different organs and tissues and it is responsible for progressive ophthalmological, neurological, and dermatological signs. In familial forms of AGel, fragments of mutated GSN are produced. These peptides show an increased propensity to assemble and precipitate into insoluble amyloid fibers. Little is known about the GSN aggregation process but based on decades of studies on better-characterized systems, we can speculate that AGel pathogenicity stems from both the toxicity of the soluble aggregates and the stress and mechanical damage caused by mature fibers [41]. Drugs preventing or slowing down such aggregation processes can limit the toxicity elicited by mutated GSN.

More than 10 single point mutations can be responsible for AGel, and D187N/Y in the second domain of GSN is the most common and better characterized. When D187 is substituted with N or Y, it causes a local destabilization that in turn triggers the aberrant proteolytic pathway, resulting in two amyloidogenic-prone fragments of 5 and 8 kDa. In order to contrast the aggregation of these two fragments, the pathological hallmark of AGel, we designed peptidomimetics built on a piperidine-pyrrolidine unnatural scaffold and highly selective for the aggregation core of G2.

When tested in ThT fluorescence assays, two of the peptidomimetics showed to be effective in slowing down and blocking the aggregation of the isolated amyloidogenic core and the entire G2 domain in a dose-dependent manner. The TEM images showed that LB-6 was able to completely inhibit the formation of fibrils. In toxicity assays performed in vivo using the nematode *C. elegans*, the aggregates formed by the amyloidogenic core showed to be toxic for the worms, as shown by the reduction in their pumping rate. When the peptidomimetics were added, LB-5 and LB-6 rescued the vitality of the worms to a similar extent and were not toxic. In contrast, LB-7 showed intrinsic toxicity and could not reduce the toxicity of the aggregates.

Our data support the feasibility of the exploited approach, showing that once the sequences of the amyloidogenic trigger, and those of possible interactors, are known, it is possible to design molecules able to specifically inhibit the formation of the toxic species. Advanced knowledge of all the AGel forms’ underlying mechanisms and the identification of other amyloidogenic cores of the protein will lead to the design of inhibitors against the G4:G5 variants and more exotic forms of the disease, or, more in general, against other amyloidosis.

## 4. Materials and Methods

### 4.1. Synthesis of the Peptidomimetics

Both the reagents and the solvents were purchased from common commercial sources and used without additional purification. The GAC_182–192_ amyloidogenic core and LBs peptidomimetics were synthesized by microwave-assisted Fmoc/tBu-based solid phase peptide synthesis (SPPS) using an automated synthesizer (Liberty Blue, CEM). Fmoc-protected piperidine–pyrrolidine scaffold was prepared in accordance with our published procedure [30,42]. Rink-Amide resin (0.69 mmol/g loading) was used as solid support and the synthesis was carried out on a 0.1 mmol scale. All used amino acids were N-terminally Fmoc-protected, while the side chains of trifunctional amino acids were protected with orthogonal, acid labile groups. The coupling was performed using 5 equivalents (eq) of the protected amino acid, previously dissolved in DMF to obtain a 0.2 M solution. As coupling reagents, 5 eq of DIC (0.5 M in DMF) and 5 eq of Oxyma Pure (1 M in DMF) have been used. To deprotect the Fmoc group, a solution of piperidine in DMF (20% *v*/*v*) has been applied. The coupling reaction has been accomplished at 25 °C for 120 s, followed by 480 s at 50 °C and 35 W. The Fmoc group was cleaved with a standard deprotection protocol at 75 °C, 155 W for 15 s followed by 60 s at 90 °C, 50 W. The coupling of the synthetic scaffold was performed directly on resin, using as coupling system HOBt/HBTU/DIEA (1.5/1.5/eq) and running it overnight at room temperature. Finally, the peptidyl-bound resins were cleaved with a mixture of TFA/phenol/H_2_O/thioanisole/TIPS (84:5:5:5:1 *v*/*v*/*v*/*v*) for 2 h under magnetic stirring. After the work-up, the so obtained crudes were purified by RP-HPLC on a C18-Classic 10 μ 250 × 21.2 mm ID (Adamas, Sepachrom, Rho (MI), Italy). Compound purity was verified by analytical RP-HPLC on a Gemini-NX 5μ C18 110A 150 × 4.6 mm (Phenomenex, Torrance, USA, CA) (Figure S4). Mass spectra were acquired on Fisons MD800 spectrometer and electrospray ion trap on a Finnigan LCQ advantage Thermo-spectrometer (Figure S4).

### 4.2. Characterization of the Compounds

For the aggregation and toxicity analysis, the LB compounds were dissolved in ddH_2_O to a concentration of 10 mM (LB-6 and LB-7) or in 10% acetonitrile at a concentration of 5 mM (LB-5). Due to its high tendency to form aggregates, GAC_182–192_ was dissolved in 10 mM NaOH to a concentration of 1 mM just before the beginning of the experiments and promptly diluted to the desired concentration in the assay solution.

### 4.3. Expression and Purification of G2_D187Y_

The isolated G2 domain containing the D187N substitution was produced using the protocols already reported in [21,43].

### 4.4. Tht Assays

Aggregation kinetics were followed using a Thioflavin-T (ThT) fluorescence assay, based on the increase of the fluorescence signal of ThT when bound to β sheet-rich structures [44]. The GAC_182–192_ peptide, at a final concentration of 100 µM, and the D187Y G2 isolated domain, at a final concentration of 75 µM, were incubated under continuous orbital shaking in 200 mM sodium citrate buffer pH 4.5, 100 mM NaCl and in the presence of decreasing molar ratio of LB-5, LB-6 and LB-7, at 37 °C in the presence of 20 μM ThT (100 μL solution/well). ThT fluorescence was measured every 5 min in microplate wells (Microplate Corning 3881, 96-well, low-binding, Corning Inc. Life Sciences, Acton, MA, USA) using a Thermo Scientific™ Varioskan™ LUX multimode microplate reader. The dye was excited at 448 nm, and the emission was measured at 485 nm.

### 4.5. Transmission Electron Microscopy

Samples were prepared under the same conditions as in the ThT-fluorescence assay and 5 µL of 100 µM GAC_182–192_ in 200 mM sodium citrate buffer pH 4.5, 100 mM NaCl in the presence and absence of LB-6 were adsorbed onto 300-mesh Copper carbon grids after glow-discharge at 30 mA for 30 s. After 1 min the grids were blotted with Whatman filter paper and laid on a 2% uranyl acetate solution drop for 1 min. Finally, they were air-dried. Grids were observed at the TEM Talos L120C (FEI, Thermo Fisher Scientific, Hillsboro, OR, USA) operating at 120 kV. Images were recorded with a Ceta camera 4k × 4k resolution.

### 4.6. C. elegans Studies

The Bristol N2 strain was obtained from the Caenorhabditis elegans Genetic Center (CGC, University of Minnesota, Minneapolis, MN, USA) and propagated at 20 °C on a solid Nematode Growth Medium (NGM) seeded with *E. coli* OP50 (CGC) for food. To investigate the effect of the peptide in the different aggregation states, GAC_182–192_ was dissolved at 100 µM in 200 mM sodium citrate buffer, pH 4.5, containing 100 mM NaCl and the aliquot was withdrawn immediately after the dissolution (Monomer) and 48 h after the incubation at 37 °C (Aggregate). Samples were then diluted in 10 mM phosphate buffered saline, pH 7.4, to the final concentration of 20 µM and administered to *C. elegans* (50 worms/50 µL) as already described [23]. Control worms were treated in the same experimental condition with 10 mM phosphate buffered saline, pH 7.4 (50 worms/50 µL) (Vehicle). After 2 h on orbital shaking, worms were transferred onto fresh NGM plates seeded with OP50 *E. coli*. The pharyngeal pumping rate, measured by counting the number of times the terminal bulb of the pharynx contracted over a 1 min interval (pumps/min), was scored 2 and 24 h later.

The effect of peptidomimetics was investigated by administering them to worms at 2 or 20 µM and determining the pharyngeal activity 24 h later. The protective activity of the peptidomimetics was studied by incubating GAC_182–192_ (100 µM) in a 200 mM sodium citrate buffer, pH 4.5, containing 100 mM NaCl, for 48 h at 37 °C in the absence or presence of 10 µM LB-5, LB-6 or LB-7. At the end of incubation, samples were diluted five times and 20 µM GAC_182–192_ + 2 µM peptidomimetic was administered to worms (50 worms/50 µL). Control worms were treated with 20 µM GAC_182–192_, 2 µM LB-5, LB-6, or LB-7 alone, or 200 mM sodium citrate buffer, pH 4.5 (50 worms/50 µL).

### 4.7. Statistical Analysis

The data were analyzed using GraphPad Prism 9.0 software (San Diego, CA, USA) by one-way or two-way ANOVA, and Bonferroni’s post hoc test. A *p*-value < 0.05 was considered significant.

## Supplementary Materials

The following supporting information can be downloaded at: https://www.mdpi.com/article/10.3390/ijms232213973/s1.
