# Supplementary material for: Rational Design of a Peptidomimetic Inhibitor of Gelsolin Amyloid Aggregation
Source: Int J Mol Sci. 2022 Nov 12;23(22):13973. doi: 10.3390/ijms232213973 (PMC9698219; doi:10.3390/ijms232213973)
Supplement: Supplementary file 1 [file ijms-23-13973-s001.zip › ijms-2028919-supplementary.pdf]

Rational design of a peptidomimetic inhibitor of gelsolin amyloid aggregation

Michela Bollati[1], Kaliroi Peqini[2], Luigi Barone[2], Carmina Natale[3], Marten Beeg[3], Marco Gobbi[3], Luisa Diomede[3], Michelangelo Trucchi[1], Matteo de Rosa[1]* and Sara Pellegrino[2]*

[1] Institute of Biophysics, National Research Council (IBF-CNR), c/o Department of Biosciences, University of Milano, Via Celoria 26, 20133 Milano, Italy

[2] Department of Pharmaceutical Science, "A. Marchesini" General and Organic Chemistry Section, University of Milano, Via Venezian 21, 20133 Milano, Italy

[3] Department of Molecular Biochemistry and Pharmacology, Istituto di Ricerche Farmacologiche Mario Negri IRCCS, Via Mario Negri 2, 20156 Milano, Italy

Supplementary figures:

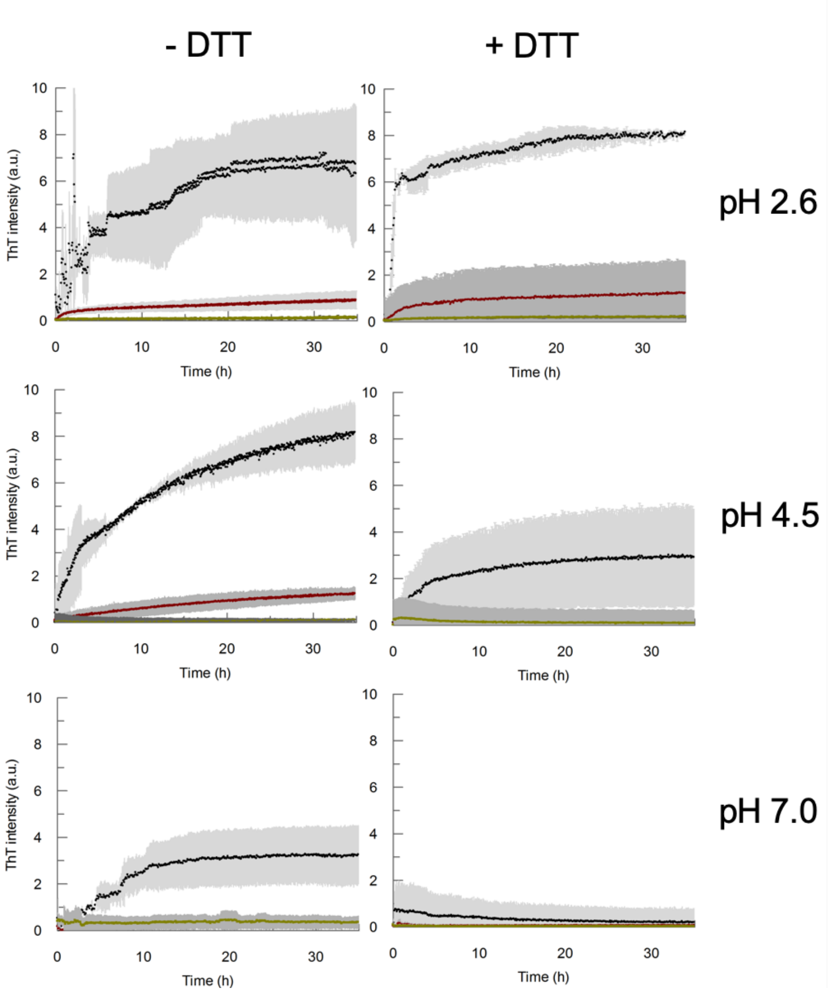

Figure S1: *Representative curves of ThT fluorescence assays showing* GAC$_{182-192}$ *aggregation under different conditions. The concentration of* GAC$_{182-192}$ *was varied between 25(green trace) and 100 µM (black trace), in 100 mM NaCl and 200 mM Sodium Acetate pH 2.6, or 200 mM Sodium Citrate pH 4.5, or 200 mM HEPES pH 7.0; Both in the presence and absence of 1 mM DTT.*

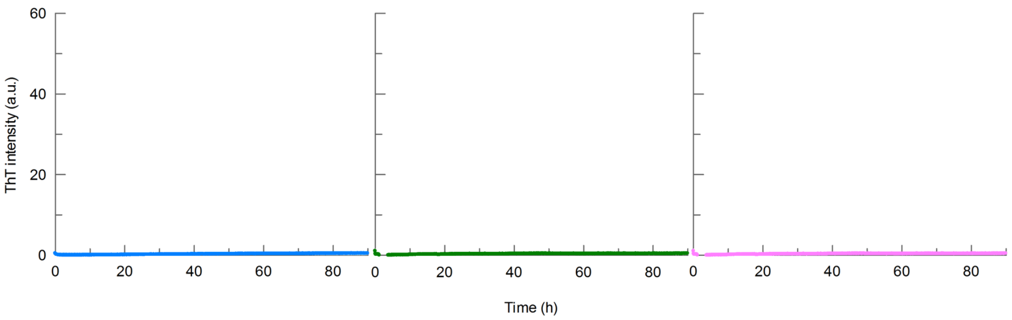

Figure S2: *ThT fluorescence of the LB peptidomimetics (LB-5 blue trace; LB-6 green; LB-7 magenta) under the same conditions used for* GAC$_{182-192}$ *aggregation assay and at the highest concentration tested. Y axis scales as in figure 2.*

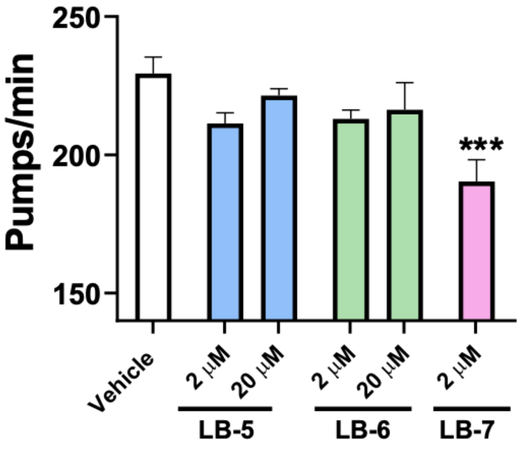

Figure S3: *Effect of peptidomimetics on the pharyngeal function of C. elegans. LBs were administered to worms at 2 or 20 μM in 200 mM sodium citrate, pH 4.5, containing 100 mM NaCl (50 worms/50 μL). Control worms were treated with 100 mM sodium citrate, pH 4.5 alone (50 worms/50 μL) (Vehicle). The pharyngeal activity was determined 24 h after treatment. Data are mean ± SE (N=10). ***p<0.001 vs Vehicle, one-way ANOVA, and Bonferroni's post hoc analysis.*

$GAC_{182\text{-}192}$

HPLC chromatogram

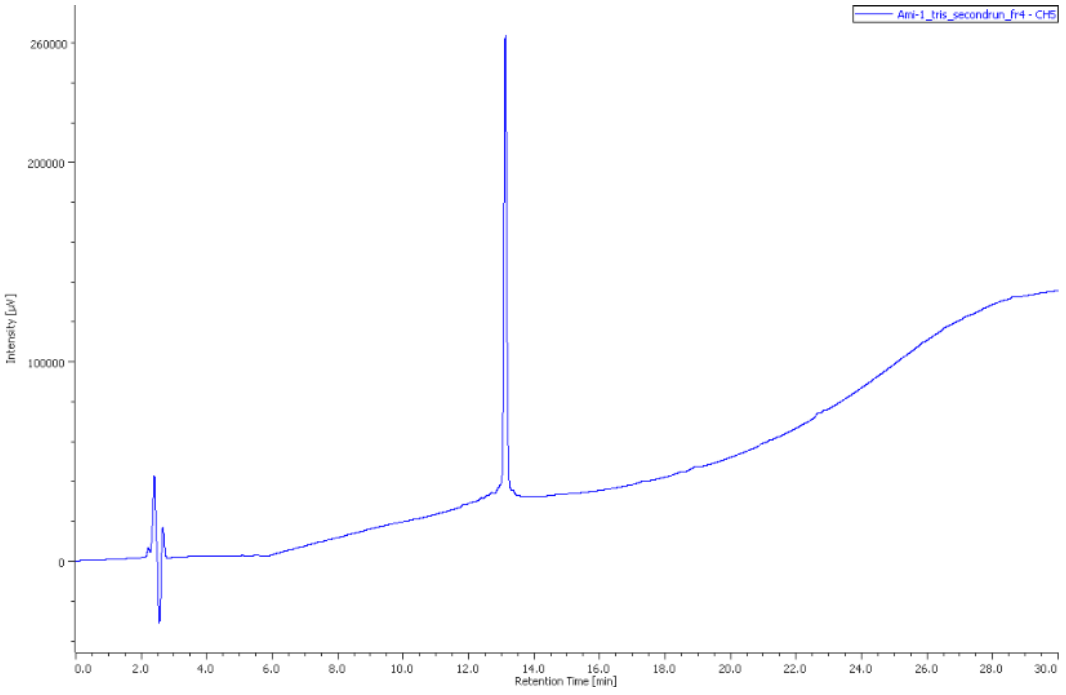

ESI/MS spectrum

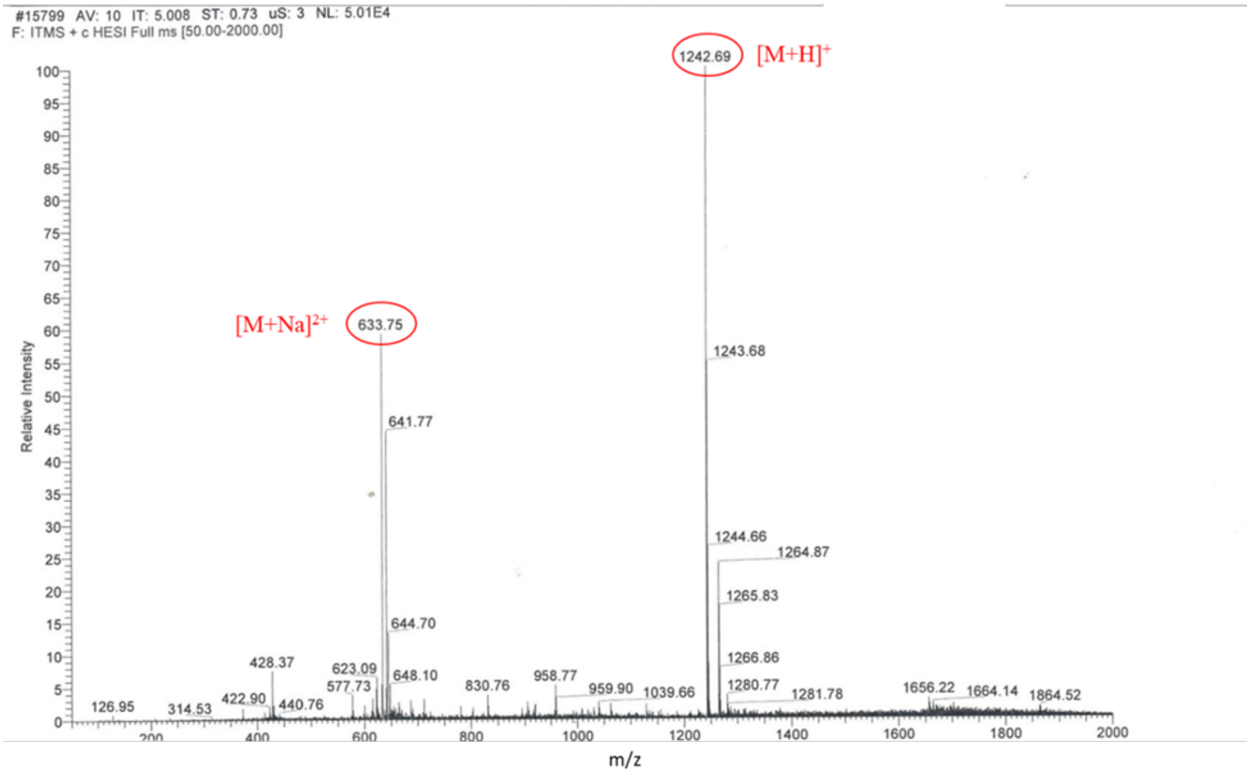

LB-5

HPLC chromatogram

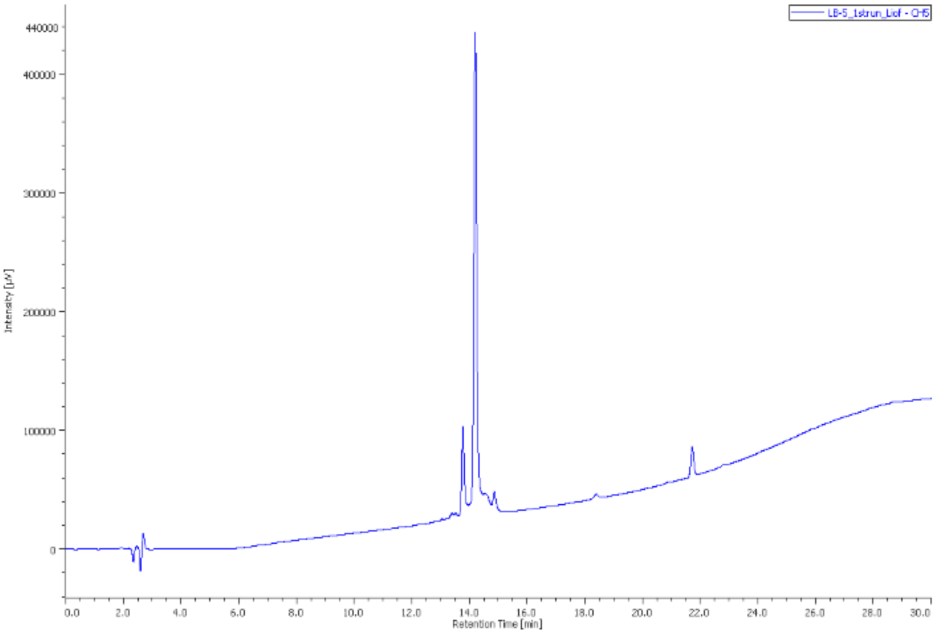

ESI/MS spectrum

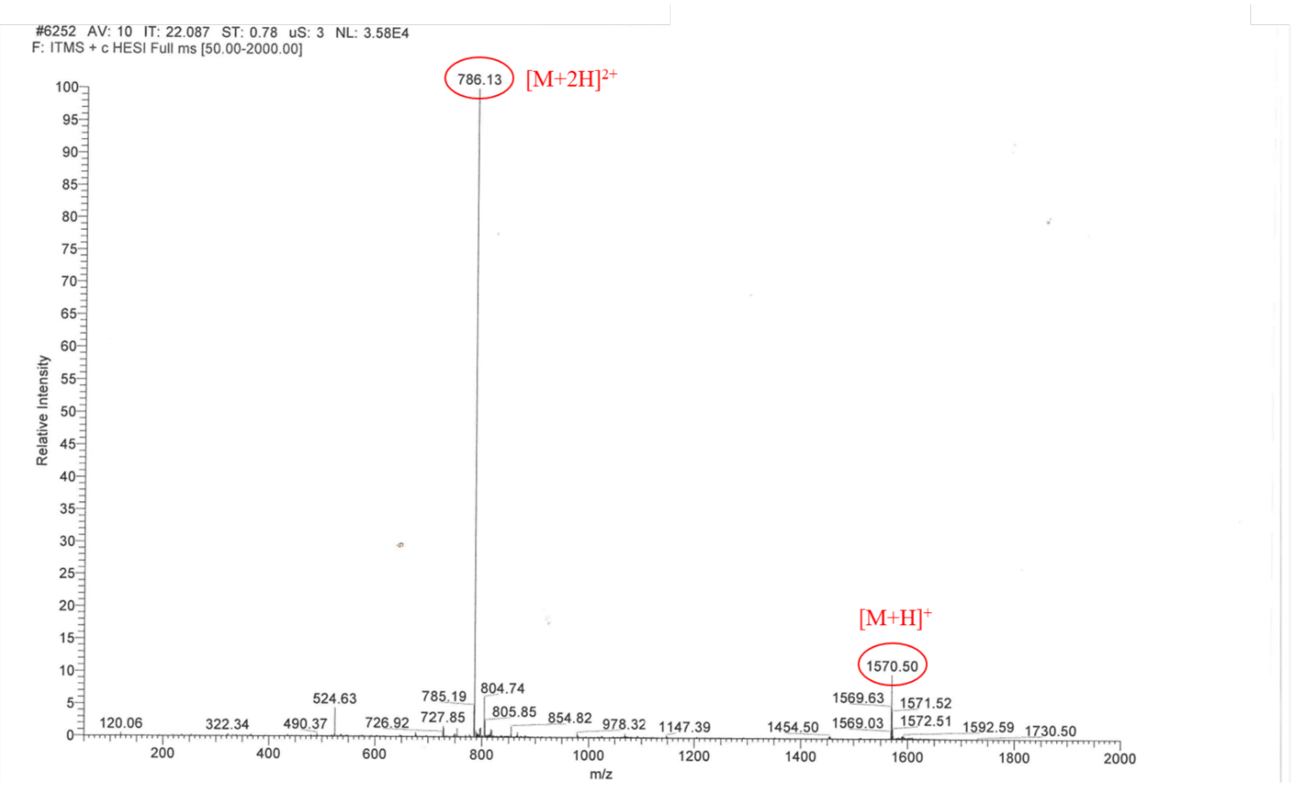

LB-6

HPLC chromatogram

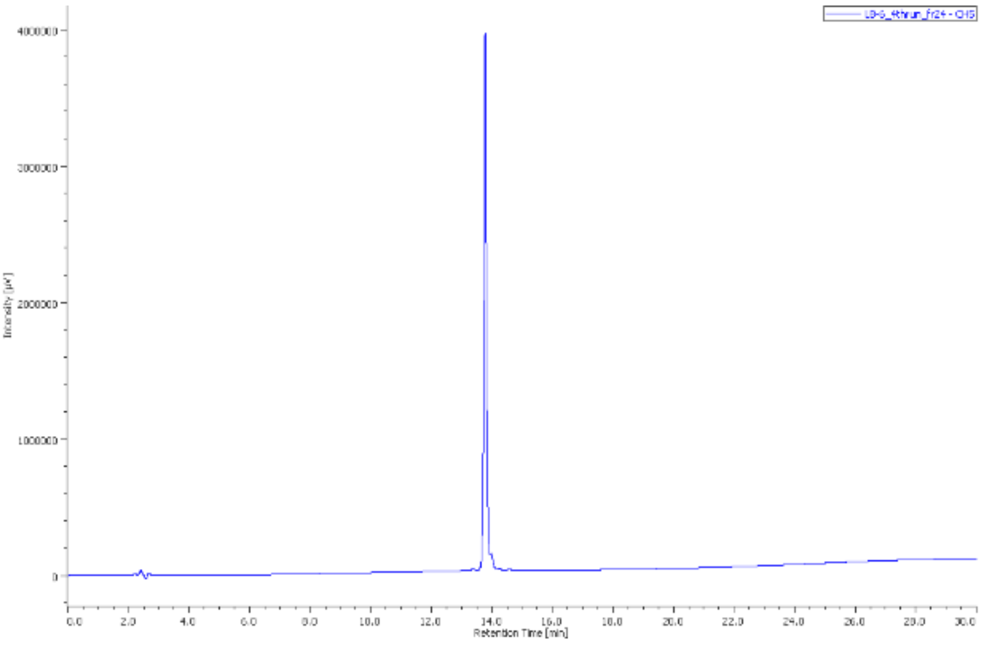

ESI/MS spectrum

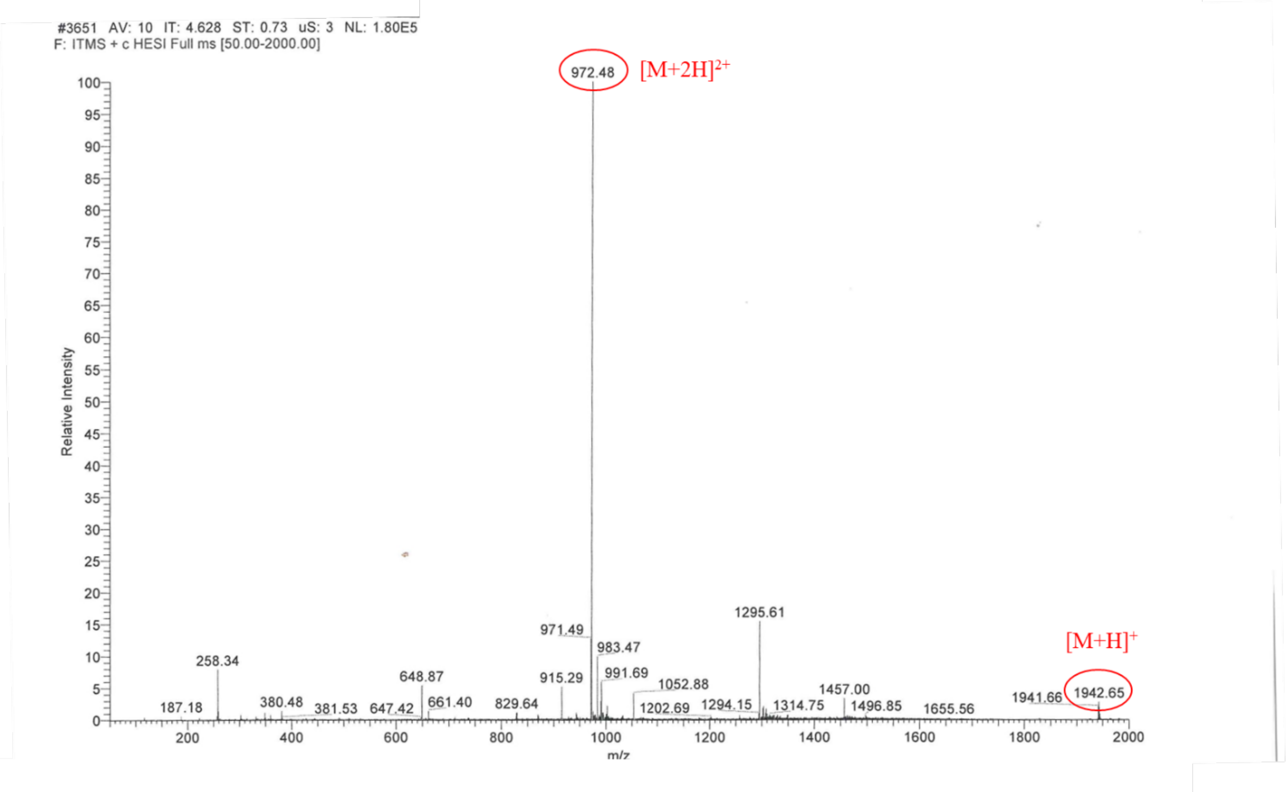
#3651 AV: 10 IT: 4.628 ST: 0.73 uS: 3 NL: 1.80E5
F: ITMS + c HESI Full ms [50.00-2000.00]
972.48
[M+2H]2+
[M+H]+
1942.65
1295.61
971.49
983.47
991.69
1052.88
258.34
648.87
915.29
1457.00
1941.66
187.18
380.48
381.53
647.42
661.40
829.64
1202.69
1294.15
1314.75
1496.85
1655.56
Relative Intensity
m/z

HRMS

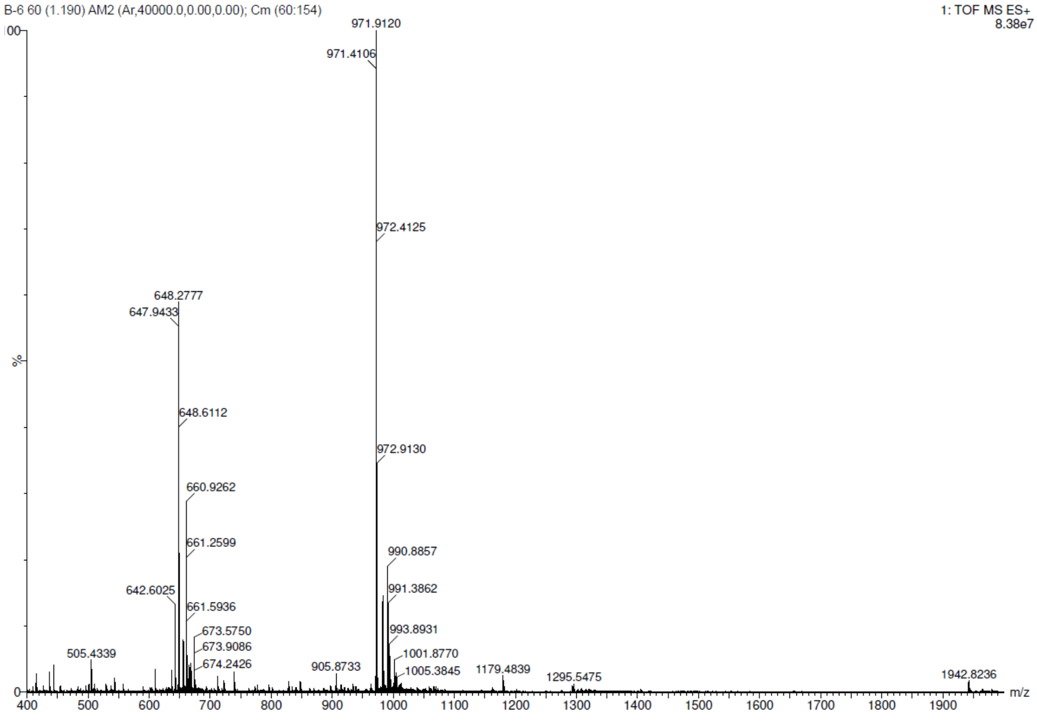
B-6 60 (1.190) AM2 (Ar,40000.0,0.00,0.00); Cm (60:154)
1: TOF MS ES+
8.38e7
971.9120
971.4106
972.4125
648.2777
647.9433
648.6112
972.9130
660.9262
661.2599
990.8857
991.3862
642.6025
661.5936
993.8931
673.5750
673.9086
674.2426
1001.8770
1005.3845
505.4339
905.8733
1179.4839
1295.5475
1942.8236
%
m/z

LB-7

HPLC chromatogram

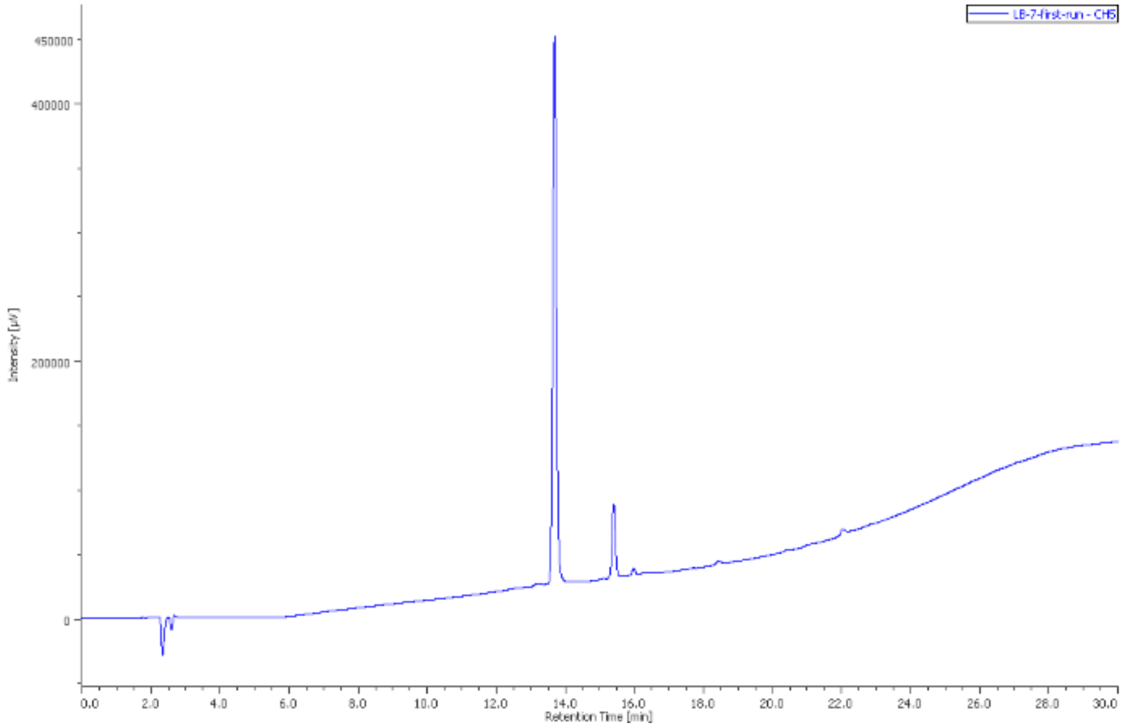

ESI/MS spectrum

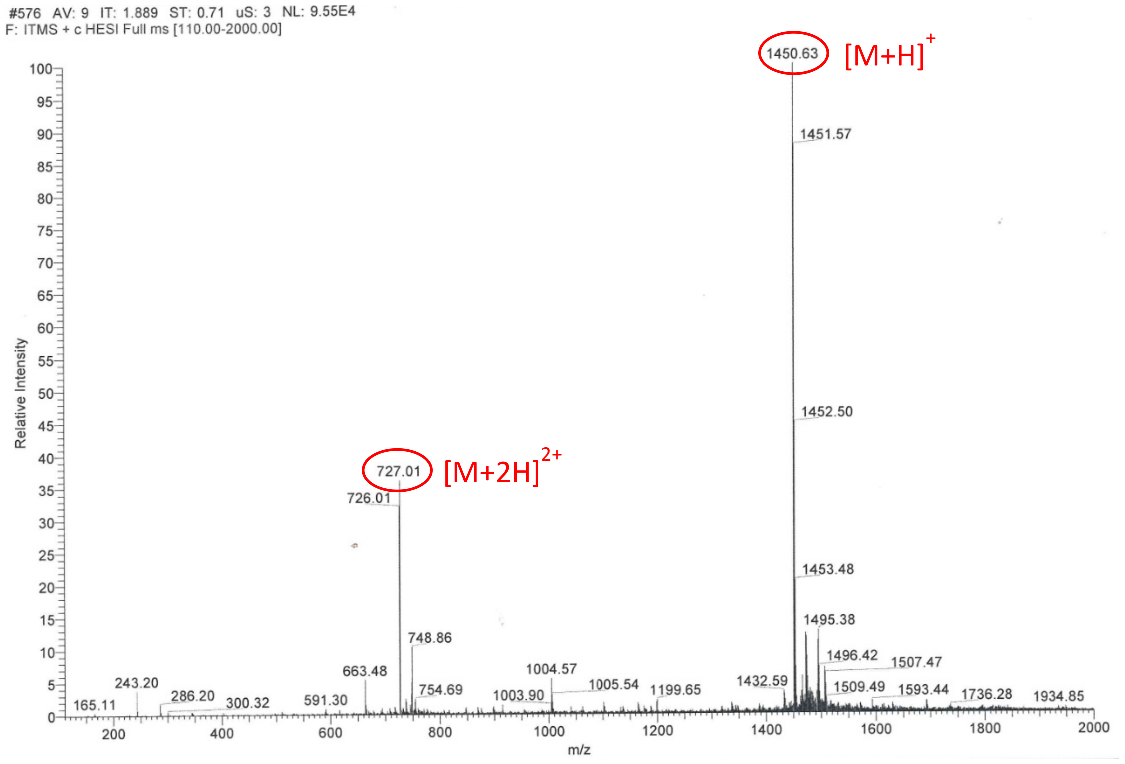

Figure S4: *RP-HPLC chromatograms, GC-MS and ESI spectra of purified GAC$_{182-192}$, LB5, LB6 and LB7. HRMS of LB6.*
